# Statin Medication Improves Five-Year Survival Rates in Patients with Head and Neck Cancer: A Retrospective Case-Control Study of about 100,000 Patients

**DOI:** 10.3390/cancers15123093

**Published:** 2023-06-07

**Authors:** Jonas Wüster, Max Heiland, Susanne Nahles, Robert Preissner, Saskia Preissner

**Affiliations:** 1 Department of Oral and Maxillofacial Surgery, Charité—Universitätsmedizin Berlin, Corporate Member of Freie Universität Berlin, Humboldt-Universität zu Berlin, and Berlin Institute of Health, Augustenburger Platz 1, 13353 Berlin, Germany; jonas.wuester@charite.de (J.W.);; 2 Institute of Physiology and Science-IT, Charité—Universitätsmedizin Berlin, Corporate Member of Freie Universität Berlin, Humboldt-Universität zu Berlin, and Berlin Institute of Health, Philippstr. 12, 10115 Berlin, Germany; robert.preissner@charite.de

**Keywords:** statins, head and neck cancer, HNSCC, OSCC, survival, real-world data

## Abstract

**Simple Summary:**

Usually: statins are prescribed to lower cholesterol levels. Besides, various studies showed that statins have beneficial effects on cancer prevention and treatment. We investigated the effect of statin medication in patients with head and neck cancer in a real-world cohort from a federated network of more than 80 healthcare organizations. We created two cohorts diagnosed with head and neck cancer, with similar age, sex, and risk factors like alcohol and nicotine. Cohort I received statins and cohort II did not. Both cohorts contained about 50,000 patients. We performed a survival analysis and found five-year survival was to be significantly higher for cohort I and a lower risk of death, respectively. As our analysis was conducted retrospectively, the results need further clinical research to be confirmed.

**Abstract:**

Introduction: The overall survival among head and neck cancer patients is still low, even in a time of new therapy regimes. Regarding cancer patients’ survival, statin use has already proven to be associated with favorable survival outcomes. Our objective was to investigate the influence of statin medication on the overall survival of head and neck cancer patients. Methods: Retrospective clinical data of patients diagnosed with head and neck cancer (International Classification of Diseases codes: C00–C14) were retrieved from a real-world evidence database. The initial cohort was divided into patients with statin medication, who were assigned to building cohort I, and subjects without statin medication, who were assigned to cohort II, both matched by age, gender, and risk factors (nicotine and alcohol abuse/dependence). Subsequently, Kaplan–Meier and risk analyses were performed, and odds and hazard ratios were calculated. Results: After matching, each cohort contained 48,626 patients (cohort I = females: 15,409; (31.7%), males 33,212 (68.3%); mean age ± standard deviation (SD) at diagnosis 66.3 ± 11.4 years; cohort II = females: 15,432; (31.7%), males 33,187 (68.2%); mean age ± standard deviation (SD) at diagnosis 66.4 ± 11.5 years). Five-year survival was found to be significantly higher for cohort I, with 75.19%, respectively 70.48% for cohort II. These findings were correlated significantly with a risk of death of 15.9% (cohort I) and 17.2% (cohort II); the odds ratio was 0.91 (95% CI: 0.881–0.942) and the hazard ratio 0.80 (0.777–0.827). Conclusions: The results indicate that the five-year survival of head and neck cancer patients is significantly improved by statin medication. As this study was conducted retrospectively, our data must be interpreted with caution, especially since other potential influencing factors and the initial tumor stage were not available.

## 1. Introduction

Head and neck cancers (HNC) are the seventh-most-common tumors worldwide, including all epithelial malignancies of the oral cavity, pharynx (subdivision into naso-, oro-, and hypopharynx), larynx, nasal cavity, paranasal sinus, and salivary glands [1,2,3]. Apart from HNC related to heavy tobacco and/or alcohol consumption [2,4], HPV-associated HNC has increased, especially HPV-positive oropharyngeal squamous cell cancer (OPSCC) among younger people [5]. Lately, due to improved radiotherapy, the introduction of concurrent radio-sensitizing systemic therapy and definitive radiotherapy/chemoradiotherapy, the survival of patients with HNC, particularly in HPV-positive OPSCC patients, has increased noticeably [2,5].

With their lipid-lowering characteristics, statins are often used to reduce cholesterol blood levels in heart disease(s) and stroke prevention [6,7]. This effect is achieved through competitive inhibition of the enzyme, 3-hydroxy-3-methylglutaryl-coenzyme A reductase (HMG-CoA reductase; HMGCR), the key enzyme of the cholesterol synthesis pathway [7]. The structural similarity between the acid form of statins and HMG-CoA as the natural substrate of HMGCR allows the inhibition of this process [7]. Normally, the conversion of 3-hydroxy-3-methylglutaryl-coenzyme A (HMG-CoA) into mevalonate is catalyzed by HMGCR [7]. Decreasing mevalonate levels result in a higher expression of low-density lipoprotein (LDL) receptors on the cell surface and consequently, in an increased LDL catabolism [7,8]. In addition, (clinical) data have revealed that some statins are capable of inhibiting the synthesis of LDL in the liver through the prevention of very-low-density lipoprotein (VLDL) synthesis. Furthermore, some statins increase the level of high-density lipoprotein (HDL) [7,9]. Regarding the influence of statins on cancer in general, recent data suggest suppression of tumor growth, metastasis, and induction of apoptosis for statins [10,11,12]. Moreover, metabolic modulation in tumors has been found to be induced by statins through inhibition of monocarboxylate transporter function [13]. Furthermore, chemopreventive effects on different solid tumors have been detected, which are probably achieved via arresting cell cycle progression, inducing apoptosis, inhibiting angiogenesis, and immunomodulation [11]. Additionally, statins may disrupt the crosstalk of cancer cells and the tumor microenvironment, which inhibits tumor progression [14,15]. Various studies have examined the in vitro effect of statins on head and neck cancer squamous cell carcinomas (HNSCC); here, statins have proven to be (dose-dependent) cytotoxic to HNSCC cells and to have reduced cell viability to less than 50% in HNSCC cell lines [16,17,18,19,20]. Recent in vitro studies suggest that cell cycle regulation and apoptosis follow persistence in the G0/G1 phase of HNSCC cells [21,22]. Clinical data on the influence of statins in cancer patients exist, e.g., for pancreatic cancer [23], lung cancer [24], and gynecological cancer [15], but data for statin use and the potential influence in HNC patients’ survival are still rare. Only a few clinical studies have focused on statin use and the potential influence on survival outcomes in HNC. Patients with hyperlipidemia and statin use at the time of diagnosis showed improved overall and cancer-specific survival compared to patients with hyperlipidemia and not taking a statin, as well as patients with hyperlipidemia and no statin use [25]. For HPV-negative HNSCC, Lebo et al. showed that patients with statins had better overall and disease-specific survival than those who were not taking a statin at or before cancer diagnosis [26,27]. Getz et al. confirmed the potential beneficial effect of statin medication on overall survival for patients suffering from HNSCC [28]. Here, a subdivision was made to examine the effect of statins on HPV-negative and HPV-positive tumors separately, revealing new findings: a protective association between statin use and disease-specific death and recurrence that was restricted to HPV-positive patients [28].

Aiming to add further insights to the relationship between statins and HNC, this study investigates the impact of statin medication in HNC patients on the clinical outcome (five-year survival) in a large case-control study. In this context, the TriNetX Global Health Research Network (TriNetX, Cambridge, MA, USA), a real-world database, was selected to gain data on this subject.

## 2. Materials and Methods

### 2.1. Ethics Statement

By the local legislation and institutional requirements, no ethical review/approval was required, due to the retrospective nature of the study and the de-identification of the data. Likewise, written informed consent was not required following the national legislation and the institutional requirements.

### 2.2. Data Acquisition, Inclusion and Exclusion Criteria, and Patient Matching

The TriNetX Global Health Research Network provides access to medical records from more than 80 healthcare organizations (HCOs) in 30 countries, enabling the collection and exchange of longitudinal clinical data between contract research institutes and pharmaceutical companies. At the time of data acquisition, electronic medical records of more than 250 million individuals were collected and available for statistical analysis, as implemented in previous studies [29,30]. The TriNetX database was searched for individuals who were diagnosed with HNC (International Classification of Diseases (ICD)-10 codes C00–C14 5 to 20 years before the access date (27 January 2023)). To be included, patients’ medical records had to cover at least five years (1825 days) of follow-up after visiting the HCO for an inpatient encounter. Medical records older than 20 years were not included. Patients with statin medication were assigned to cohort I (subjects with ICD-10 codes C00–C14 and medication of Rosuvastatin, Simvastatin, Fluvastatin, Pravastatin, Lovastatin, Atorvastatin, and Pitavastatin). Further, cohort II was formed of subjects who were diagnosed with ICD-10 codes C00–C14 and no history of statin medication. As in previous studies, one-to-one matching was performed for age, gender, and tobacco and/or alcohol abuse (ICD-10: Z87.891, F10.1, or F10.2) to receive randomized conditions as closely as possible by obtaining cohorts with similar covariate distributions [29,30].

### 2.3. Data Analysis

**Figure 1:**
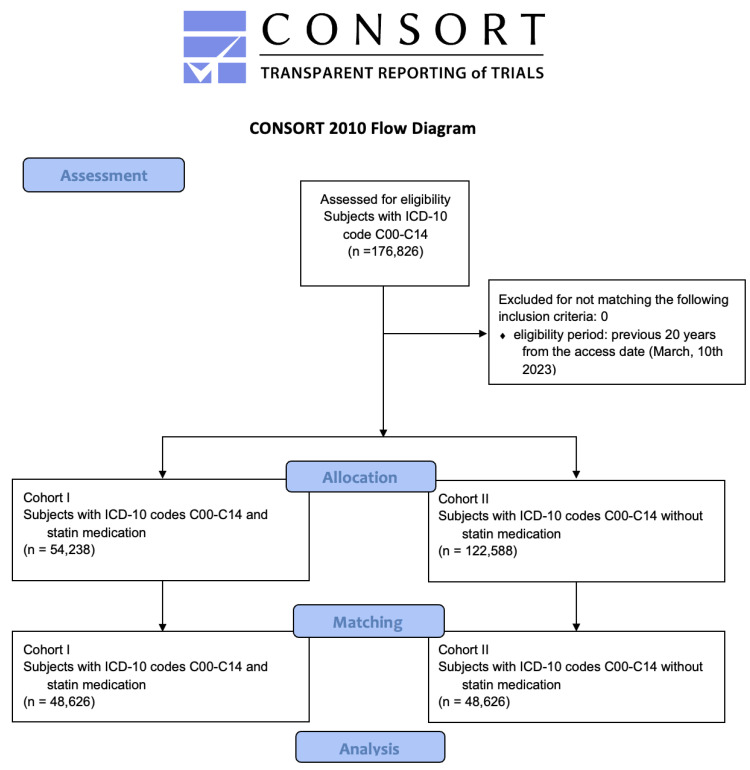
Modified CONSORT flowchart.

The primary outcome was defined as “death” with subsequent calculation of Kaplan–Meier survival analysis, Cox proportional hazards regression, risk ratios (RR), odds ratios (OR), and hazard ratios (HR) for each cohort. Data analysis was limited to a period of 5 years after the first HNC diagnosis, as patients are regarded as healed in case of absence/no recurrence of HNC or metastases within the defined period. Statistical analysis was performed using the log-rank test, whereby the probability level for statistical significance was set at 5% (*p* = 0.05). Before matching, 136,755 patients were assigned to cohort II. Subsequently, with one-to-one matching, the same number of patients as in cohort I (n = 48,626 patients) were assigned to cohort II (Figure 1).

## 3. Results

### 3.1. Assessment, Allocation, and Matching

Overall, 176,826 patients from 73 HCOs met the inclusion criteria (ICD-10 codes C00-C14) and could be retrieved from the database. Firstly, cohort I was defined by the use of statins, which led to a cohort of 54,238 patients (females: 16,966 (31.3%), males 7309 (68.7%), mean age ± standard deviation (SD) 67.7 ± 11.2 years). All patients who met the inclusion criteria and had no history of statin medication were assigned to cohort II, resulting in a cohort of 122,588 patients (females: 37,309 (33.0%), males 82,100 (67.0%), mean age 58.5 ± 16.7 years). Subsequently, using propensity score matching, 48,626 patients were assigned to each cohort, which led to cohorts as follows:

Cohort I with 15,409 (31.7%) females, 33,212 (68.3%) males and a mean age at diagnosis of 66.3 ± 11.4 years. Within the cohort, I, 5710 patients (11.7%) with ICD-10 code Z87.891, 2088 patients (4.3%) with ICD-10 code F10.1, and 1689 patients (3.5%) with ICD-10 code F10.2 were found.

Cohort II with 15,432 (31.7%) females, 33,187 (68.2%) males and a mean age of 66.4 ± 11.5 years. Here, 5596 patients (11.5%) with ICD-10 code Z87.891, 2047 patients (4.2%) with ICD-10 code F10.1, and 1658 patients (3.4%) with ICD-10 code F10.2 were found. The patient characteristics of cohort I and cohort II, before and after matching, are listed in Table 1.

### 3.2. Patient Survival

**Figure 2:**
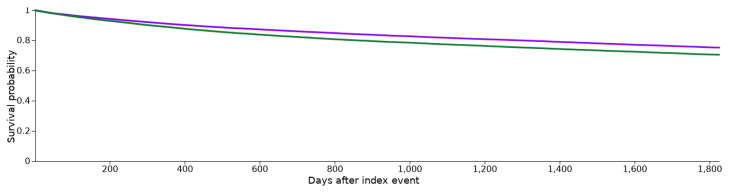
Kaplan–Meier survival curves for both cohorts. Cohort I (ICD-10 codes C00-C14 and statin medication; purple) and cohort II (ICD-10 codes C00-C14 without statin medication; green).

**Figure 3:**
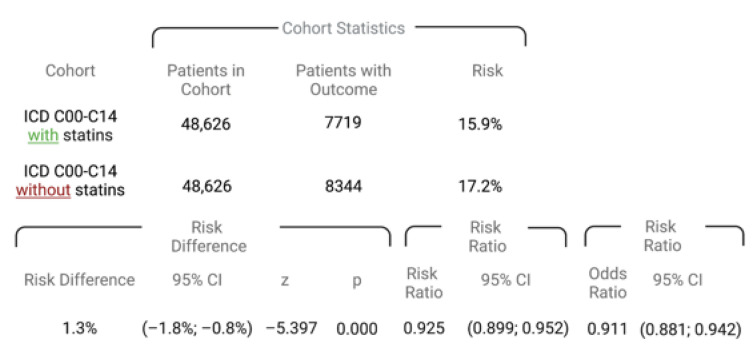
Risk of death, risk ratios, and odds ratio of cohort I (ICD codes C00–C14 with statin use) and cohort II (ICD codes C00–C14 without statin use).

During the five-year observation period after the initial diagnosis of HNC, 7719 patients in cohort I and 8344 patients in cohort II died. These findings correlate with a risk of death of 15.9% (cohort I) and 17.2% (cohort II). The survival probability at the end of the time window was 75.19% for cohort I and 70.48% for cohort II, as seen in Figure 2. The related risk ratio (RR) was 0.925 (95% confidence interval (CI): 0.899–0.952), and the odds ratio (OR) and hazard ratio (HR) were 0.991 (95% CI: 0.881–0.942) and 0.801 (95% CI: 0.777–0.827) (Figure 3).

## 4. Discussion

The present study investigated the relationship between statin medication on the five-year survival rate in HNC patients. To the best of the authors’ knowledge, this study was the first to address this question by evaluating data retrospectively in larger cohorts. Our data indicate considerable benefits regarding the survival probability for HNC patients with statin medication compared to HNC patients without statin medication.

Recently, a growing interest in statins has arisen since statin use and its beneficial influence on cancer survival outcomes have been evaluated in previous studies [31,32,33]. In this context, most studies have revealed higher survival rates for patients with statin use, regardless of the cancer type [31,32,33,34,35]. Our retrospective study focused on patients with pre-diagnostic statin use that continued during the post-diagnostic stage, which makes the study comparable to the study design of most other studies [36,37] that have dealt with the influence of statins on cancer. Nevertheless, other findings suggest that statin use even after cancer diagnosis seems to be related to reducing overall and cancer-specific mortality [38], or even reveal that statin users after diagnosis had higher overall survival than those before diagnosis and current users [24]. Since this observation refers to patients with lung cancer, further studies might investigate whether the date of statin exposure and the influence on survival also applies to HNC patients. Besides statins, another effective cholesterol inhibitor has come into focus: ezetimibe, which is recommended in the current guidelines and increasingly used for the treatment of hypercholesterinemia, alone or in combination with statins, in the prevention of cardiovascular disease events [39,40,41]. Ezetimibe achieves its cholesterol-lowering effect by blocking the sterol transporter Niemann–Pick C1-Like 1 (NPC1L1), a key regulator of intestinal cholesterol uptake [39,42]. The latest in vitro and in vivo studies have shown that ezetimibe is capable of inhibiting prostate cancer [43] and pancreatic cancer, and protects against colitis-associated tumorigenesis [44]. Other cholesterol-lowering drugs, such as proprotein convertase subtilisin/kexin type 9 (PCSK9) inhibitors, seem to potentiate immune checkpoint therapy in cancer patients [45] and might even be used as a potential future therapeutic target in personalized cancer medicine [46]. Nevertheless, available studies still lack data about rarely used cholesterol-lowering medication. Therefore, further studies are required to investigate the influence of such medication on the survival of HNC patients.

Regarding HNC, our findings provide first clinical data on statin use and the prognostic value in HNC in a large cohort, and lead to the conclusion that statin use in HNC patients correlates with higher five-year survival. These findings support previous studies on the relationship between statin use and HNC [25,26,27,28]. In 2018, the first connection of statin use and improved overall and disease-specific survival in HPV-negative HNSCC patients, when compared to those who were not taking a statin at or before cancer diagnosis, was shown [27]. Getz et al. confirmed these findings for statin use and improved HNSCC overall survival and further compared the influence of statin medication on the outcome of HPV-negative and -positive HNSCC. Here, an inverse association between statin use and cancer-specific death was seen only in the HPV-positive cohort, which might be of interest in further studies. Since our study did not merely focus on HNSCC, but also on all HNC, the HPV-status might be negligible.

Additionally, our data support the results of various in vitro studies on the effect of statins on HNSCC cells [21,22,47] and allow translation into a clinical context. However, the obtained results need to be cautiously interpreted, as limitations of this study exist. Specifically, the TriNetX database was used to search for subjects with diagnoses (ICD-10 codes C00–C14), which presupposes the correct classification of the malignant neoplasia in the head and neck. Moreover, patients suffering from different subtypes, or even rare entities of HNC might be included in this study, leading to a certain risk of confounder bias. Furthermore, staging following the Union for International Cancer Control (UICC), clinical, histological, and molecular features, as well as the applied therapy have not been considered, despite the well-known influence on the probability of patients’ survival [48,49,50,51,52]. Statins were regarded as one group, despite different potencies and dosages. Information about the duration of statin therapy was not available but in general, statins are administered on a long-term basis. Even though one-to-one matching was performed, a certain risk of confounder bias remained since no detailed data on tobacco use (total pack years), alcohol abuse (consumed alcohol units), race [53], secondary diagnosis [54,55], and HPV status [56,57] were available.

Nevertheless, due to the large cohorts of 48,626 patients for each group and by matching, these differences should have been levelled out to a certain extent. In addition, the quality of all data retrieved from the TriNetX database can be considered as high, as the database meets the strict requirements of the National COVID Cohort Collaborative N3C. Therefore, the beneficial effect of statin use on the survival of HNC patients presented in this study might lead to further research on this topic—and if the presented results could be confirmed, HNC treatment might benefit from the chemopreventive effect of statins.

## 5. Conclusions

Pre-diagnostic statin medication in HNC patients correlates with a higher survival probability after five years (75.19%) when compared to patients without statin use (70.48%). Accordingly, the risk of death was lower in patients with statin use (15.9% vs. 17.2%). Further research is required to confirm these findings, which might lead to new supplementary treatment options in HNC therapy.

## Figures and Tables

**Table 1:** Characteristics of cohort I and cohort II, before and after matching for age, gender, tobacco use, and alcohol abuse. Note that some patients have indeterminate gender.

	Before Matching				After Matching			
Patients (n)	Cohort I	Cohort II	*p*-Value	Standardized Mean Difference	Cohort I	Cohort II	*p*-Value	Standardized Mean Difference
**Total**	54,238	122,588	<0.001	0.582	48,626	48,626	0.189	0.008
Female	16,966 (31.3%)	37,309 (33.0%)	<0.001	0.038	15,409 (31.7%)	15,432 (31.7%)	0.874	0.001
Male	37,309 (68.7%)	82,100 (67.0%)	<0.001	0.038	33,212 (68.3%)	33,187 (68.2%)	0.863	0.001
**Mean age at diagnosis (years)**	66.7	58.5			66.3	66.4		
Standard deviation	11.2	16.7			11.4	11.5		
ICD-10 Z87.891	11,307 (20.8%)	5935 (4.8%)	<0.001	0.0492	5710 (11.7%)	5596 (11.5%)	0.254	0.007
ICD-10 F10.1	2839 (5.2%)	2935 (2.4%)	<0.001	0.149	2088 (4.3%)	2.047 (4.2%)	0.515	0.004
ICD-10 F10.2	2348 (4.3%)	2520 (2.1%)	<0.001	0.129	1689 (3.5%)	1658 (3.4%)	0.586	0.003

## Data Availability

Original data is available upon request.
